# AI-Powered Navigation System for Steering POCUS in the COVID-ICU∗

**DOI:** 10.1016/j.jaccas.2020.12.020

**Published:** 2021-02-17

**Authors:** Sameer Raina, Partho P. Sengupta

**Affiliations:** Department of Medicine, Division of Cardiology, West Virginia University School of Medicine, Morgantown, West Virginia, USA

**Keywords:** cardiovascular disease, echocardiography, imaging

**Figure:**
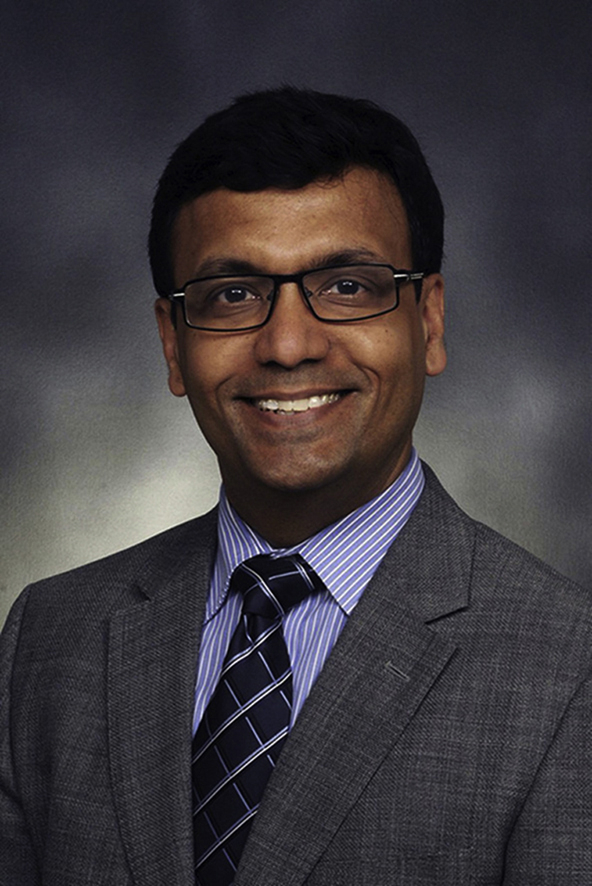

The global spread of severe acute respiratory syndrome coronavirus 2 continues with increasing numbers. As of date, more than 75 million cases of coronavirus disease-2019 (COVID-19) and more than 1.6 million deaths have been reported worldwide (1). Unfortunately, considering the contagious nature of the disease, the burden of infection and transmission among health care providers has been significant. Studies have shown that health care workers and their households have contributed a sixth of COVID-19 cases admitted to hospital (2). The increasing number of patients and limitations to the availability of personal protective equipment have created challenges in the evaluation and management of patients with COVID-19 (3). This is more so of the critically ill patients within the intensive care unit (ICU) setting who require frequent cardiopulmonary monitoring. With new data suggesting frequent cardiovascular complications especially in high-risk patients, the need for point of contact real-time data has become ever so important (4).

In this issue of *JACC: Case Reports*, Cheema et al. (5) report the successful use of novel deep learning (DL)-derived technology deployed in the COVID-19 ICU by critical care physicians with clinical experience but no formal training in ultrasound to obtain point-of-care ultrasound (POCUS) cardiac images. This case series of 5 patients provides a valuable insight into the potentials of artificial intelligence (AI)-enabled tools simplifying and finding their way into all aspects of clinical medicine. Specifically, amid the pandemic, the adoption of POCUS has been universal as first-line ultrasound examination in patients with COVID-19, which then guides the need for further downstream testing including imaging. This approach, besides being less labor intensive, nonionizing, small-sized, and flexible, helps conserve personal protective equipment and staff exposure (6). However, POCUS does come with its limitation of requiring experienced personnel who are proficient in scanning and obtaining interpretable scans. The use of AI for guiding the acquisition of high-quality cardiac ultrasound images at the bedside under prescriptive guidance and its real-world has the potential to augment bedside clinical examination and provide evidence for improving medical decision making and clinical prognostication. Cheema et al. (5), therefore, need to be commended for providing a well-illustrated proof of concept in a clinical setting in which AI-based DL technology provides real-time guidance among novice operators who lack expertise, particularly in resource-limited situations.

Over the past decade, the “DL revolution” has transformed the AI industry. The most established algorithm among various DL models is the convolutional neural network (CNN), a class of artificial neural networks that have been a dominant method in computer vision tasks. A DL model automatically and adaptively learns spatial hierarchies of features, from low- to high-level patterns using multiple layers (or building blocks), that is, convolution, pooling, and fully connected layers (7). These models can learn to discover and combine local image features (such as an edge or a color contrast) in increasing levels of abstraction to ultimately enable the prediction of an outcome. This high level of understanding from digital images and videos can help automate tasks that the human visual system can do (8). Beyond the image recognition, CNN has found use in natural language processing, recommender systems, brain-computer interfaces, drug discovery, and financial time series, a model that can predict future stock market values based on previously observed values (9,10). It also plays a key role in the safety of autonomous vehicles and their ability to account for unexpected variables while driving (11). With its multifaceted utility, there has been a recent surge of interest in CNN in image recognition in the field of medicine. CNN-based models have shown promise in the detection of skin cancer, diabetic retinopathy, and radiological diagnosis (12,13).

Despite being powerful, these models have fundamental drawbacks and limitations and by design tend to be sparing, biased, and at the mercy of the amount and type of training data that one applies. They do not encode the position and orientation of the object into their predictions and cannot also be spatially invariant to the input data. Machine vision has a limitation of understanding of 3-dimensional space, thus requiring a significant amount of time and data to train. Similar training of a human brain, for example, a sonographer performing a bedside ultrasound, would require fewer examples and less time. Other issues include overfitting when the accuracy of the model significantly underperforms in presence of a new “unseen” dataset (i.e., inability to generalize results from training data to unseen data). This is one of the main challenges in machine learning, as an overfitted model is not generalizable to “never-seen-before” data. On the contrary, underfitting occurs when a model is too simple, which makes it inflexible in learning from the dataset (14). Also, considering DL has multiple layers and performs analysis in a nonlinear manner, training time is increased. This technology needs powerful computing processing units and a cloud-based system, which can be resource limiting (15).

Although these limitations have been dodging the scalability of POCUS, its appeal continues to expand in point-of-care markets with the further miniaturization of technology, improved ease of use, lower system cost, increased portability, and greater access to training. Its use has spread beyond the hospital setting, with use by paramedics and during evacuations for the battlefield by military medics, and even in more esoteric places such as on the international space station and Everest base camp (16). Its utility in remote areas with limitations to cardiac imaging expertise has been well incorporated in telehealth models. This interpretation of images has been tested on smartphone apps, making transfer and accessibility of data more feasible (17). Another evolving solution has been real-time robot-assisted remote echocardiography followed by cardiologic consultation. This workflow involving POCUS has been found to significantly reduce the total diagnostic process time (18). Considering the increased utilization, the future estimated growth of POCUS is expected to exceed $3 billion globally by 2025, up from $1.3 billion in 2018 (19).

To make certain that such technology finds its roots in our everyday practice, we need to ensure that POCUS users have the requisite training, experience, and skillset for a seamless adoption. Legislations need to fast forward for creating required credentialing for AI-based technology. This will help streamline reimbursements while ensuring patient confidentiality, access, and maintenance of secure images, operator validation, and facility accreditation (20).

Although not undermining the prospects of a technology that may change the way we engage daily, we owe a mea culpa to the idea of replacement of human brainpower by “humanoid robots” very shortly. Our current limitation being the “shallowness of DL” itself. In its current form, AI-based POCUS is at best a primitive avatar of a navigation system that helps create a basic set of information to be interpreted by an expert health care professional. The current pandemic, however, has come as a shot in the arm and has set an excellent tailwind for a bright future for this technology.

Whether it is to help design an automated navigation system for blind people, provide balloon-based Internet platforms to underserved or difficult-to-innervate areas, or use neural network architectures to generate high-quality digital maps in resource-limited countries, AI and DL can provide a shining light in personalized care in times of limited resources and constraints (21, 22, 23). With the immense possibilities, it is only a matter of time and due diligence before AI-based health care solutions are deeply integrated to become part of our daily practice, ensuring a safer environment and improved outcomes to complex disease processes.

## Funding Support And Author Disclosures

Dr. Sengupta is a consultant for Kencor Health and Ultromics. Dr. Raina has reported that he has no relationships relevant to the contents of this paper to disclose.
